# Food acceptability and selection by astronauts on International Space Station missions informs strategies and risks for deep space exploration

**DOI:** 10.3389/fpsyg.2025.1562044

**Published:** 2025-10-30

**Authors:** Grace L. Douglas, Suzanne T. Bell, Peter G. Roma, Thomas Oswald, Millennia Young

**Affiliations:** ^1^Human Health and Performance Directorate, NASA Johnson Space Center, Houston, TX, United States; ^2^KBR, Houston, TX, United States

**Keywords:** space food system, food acceptability, food variety, menu fatigue, International Space Station, astronauts

## Abstract

Characterization of the relationship between food system acceptability and repeat consumption within the spaceflight food system may be linked to caloric intake but the impact of food acceptability and repeat consumption has not been previously systematically investigated. In this study, 15 astronauts on the International Space Station (ISS) reported acceptability of the items in one meal a week over approximately 6-month and 1-year missions. The results indicated that acceptability scores did not decrease over the mission. Astronauts limited selections to their personal favorites early in the mission and did not consume foods they did not like. Although they continued to like the foods they chose, many foods were not rated by more than one individual, supporting variability in personal preference. Intake of only liked foods from mission start may impact total variety and quantity available to each astronaut within the restricted system on ISS, which may ultimately impact total nutritional intake. It also increases the challenge for exploration missions, where food may be pre-positioned and personal preferences may not be accommodated. Astronaut comments indicated specific food types and strategies that may help improve food system acceptability for future missions.

## Introduction

The spaceflight food system is a restricted food system, which means that astronauts derive their calories and nutrition from a defined set of foods that is limited by mass, volume, and shelf-life requirements. To ensure individuals can obtain adequate calories and nutrition, the food system must provide a number of factors including food safety, nutrition, and quality, but also aspects such as menu variety and food acceptability.

Food acceptability, or liking, is an interaction between food and an individual consuming the food at any given moment of time (Maina, 2018). Characteristics of the food such as appearance, aroma, flavor, and texture all contribute to food acceptability, and there is a positive relationship between food acceptability and food intake. For example, in military samples, soldiers were more likely to choose foods again when they rated it as highly acceptable than when it was low in acceptability (de Graaf et al., 2005).

In restricted food systems the availability of foods is inherently limited, so it is necessary to consider that menu variety supports adequate nutrition and food acceptability especially as individuals are faced with repeat consumption. Menu variety is the total number of different foods made available to the individual. Over time, individuals may experience menu fatigue, where they may change their eating behavior, such as losing interest in eating the food available due to repetitive and limited variety. For individuals sustained by a restricted food system over time, menu fatigue can be particularly problematic if this decreased interest also results in insufficient caloric and nutritional intake.

Previous studies on menu fatigue or food monotony in operational environments are limited in duration and indicate that limited menu variety may result in decreased food acceptance and reduced intake within a short timeframe (Meiselman et al., 2000). Military studies with Meals Ready to Eat (MREs) resulted in a policy to limit sole use of MREs to 21 days to prevent underconsumption and weight loss associated with continued use of this limited food system (Friedl and Hoyt, 1997).

The focus of our research is on understanding the relationship between menu variety, food acceptability, and menu fatigue over time in a space food system. Current and future space exploration such as missions to Mars will be a significant human achievement. The food system is central to ensuring crew health and performance and ultimately mission success. The food system is currently considered a red risk by NASA for exploration missions, meaning that there is not currently an adequate food system strategy available that will support the constraints and timelines of these missions. Currently there are no capabilities to grow food in space at the levels needed to provide adequate caloric and nutritional intake. Therefore, astronauts rely on a shelf stable food system that fits within the mass, volume, shelf-life and other limitations of the vehicle. We next describe the current system, followed by the additional restrictions likely for space exploration, including a mission to Mars.

On the International Space Station (ISS) the diets of astronauts in the United States Operating Segment are restricted to a shared standard food system with a usage rate based on the astronauts in-mission, limited crew preference foods, and very limited fresh food supply. The majority of the food system (∼80% of the system) is part of the shared standard system that includes a consistent variety of approximately 200 different shelf stable foods and beverages packaged in lightweight flexible packaging. The food system is stored at ambient temperatures, must be easily prepared (heat, add water only), and must have a multi-year shelf life. Much of the food is produced and provisioned months in advance of a mission and arrives to the ISS prior to the astronauts who will consume it. The food system is designed to meet nutritional, quality, safety, and limited resource requirements. Foods are rated through sensory evaluation in the Space Food Systems Laboratory at the NASA Johnson Space Center (Houston, TX). Specifically, panels of volunteers evaluate the foods and rate them using a 9-pt hedonic scale (1 = Dislike Extremely; 9 = Like Extremely). Only foods rated above 6.0 are included in the system.

The standard food system is supplemented with Crew Specific Menu foods (CSM – foods chosen by each astronaut that meet spaceflight requirements; ∼20% of the system). CSM foods consist of shelf stable foods, with limited foods from International Partners (IPs), which are designated for the astronaut that chose them. Fresh foods (e.g., apples, oranges) are limitedly available to astronauts when small amounts are provided during a resupply mission.

Although only foods rated as acceptable by panels are included in the shared standard food system, specific astronauts on a mission may differ in their opinion of a specific food’s acceptability and have different preferences for foods within the standard system. To account for this, the shared food system is provisioned to provide as much variety as possible within the mass, volume, and usage rate limitations to enable astronauts some choice. This may work well if members of a crew have different preferences, but it can be a challenge if they have the same preferences. Therefore, menu fatigue, or a change in eating behavior due to the repetition of foods and limited variety, is still a concern.

ISS astronauts often comment in debriefs on the importance of menu variety and choice, and that they may tire of certain foods over their mission (∼6 months), indicating menu fatigue. However, resupply logistics and astronaut mission assignment timelines limit the ability to provide a greater percentage of CSM foods. Additionally, body mass loss is often experienced by ISS astronauts (Smith et al., 2021). The perception of food acceptability related to a restricted food system may increase in significance for future exploration missions in which food may be prepositioned (e.g., launched ahead of crew or any final crew changes). Planetary physics restricts launch windows to Mars, which may impact the ability to provision CSM foods that align with specific astronauts. Return capability may also be restricted, which may increase the importance of the acceptability of the food system to maintain nutritional intake, health, and performance for multi-year durations.

Thus, the overarching purpose of our research was to characterize food acceptability over time in astronauts on 6-or-12-month missions aboard the International Space Station. Our first objective was to determine the impact of repeat consumption on food acceptability within the current variety restrictions of the spaceflight food system. We hypothesized that menu fatigue due to repeat consumption of foods in a variety restricted system will lead to decreases in acceptability of individual foods and increased aversion to available foods over a mission. Our second objective was to identify strategies that may improve food system acceptability over time. We used a mix of qualitative and quantitative data to address these objectives.

## Methods

We collected and analyzed data from 15 astronauts (8 M/7 F) living and working on the ISS for 166–355 days to address our research questions. This study was approved per NASA IRB protocol STUDY00000107 for inclusion of human subjects.

Our first objective sought to determine the impact of repeat consumption on food acceptability within the current variety restrictions of the spaceflight food system. To answer this question a questionnaire was administered at one meal a week to capture *in situ* scoring of consumption experiences. Astronauts indicated which foods and beverages they consumed at this meal and then rated each food or beverage on its overall acceptability using a 9-pt hedonic scale (1 = Dislike Extremely; 9 = Like Extremely). Participants then provided open-ended feedback regarding food context, attributes of the food, and the meal. The repetition in survey administration was intended to target repeat consumption ratings of foods prior to menu fatigue, where ratings may decrease to a point where an astronaut might stop eating those foods and begin eating other available foods or limiting their choices to their favorites. Astronauts also participated in a post-mission debrief interview.

We analyzed the data using a mixed method approach. Descriptive statistics and visualizations were used to quantitatively summarize food acceptability ratings over time. The primary goal was to look for trends in the trajectories of ratings of the food items over time – both within and between individuals – to determine if there were substantial decreases in acceptability across all foods. The number of times a particular item was scored was used as an indicator of frequency of consumption (i.e., preference). Variety was operationalized as the overall number of unique items scored by an astronaut. We assessed whether individuals were more likely to have more observations on food items that are rated higher, and whether those who rated foods higher had more or less overall variety. Summary statistics for each food item (number of ratings, number of astronauts who rated, mean, standard deviation, min, and max rating) and astronaut (number of ratings, number of unique items, mean, standard deviation, min, and max ratings) were estimated. Associations between ratings, number of items, and mission length were analyzed by estimating correlations between mission summaries of variety (number of unique items scored), mission duration, and average numeric rating. Repeated measures correlation (Bakdash and Marusich, 2017) was used to estimate correlation between the number of times a crewmember rated a food item and the average rating adjusting for the repeated measures within crew across each unique rated menu item. Mixed models including subject-specific random effects to address the repeated measures were used to explore effect of time and repeat consumption on the numeric ratings. Analyses were run in R v4.5.0 with packages rmcorr_0.7.0 and nlme_3.1-168.

Our second objective sought to identify possible strategies that may be used in the design of the food system to reduce menu fatigue and improve acceptability over time, even within a limited variety system. To answer this question we engaged in a reflexive thematic analysis of three open-ended comment prompts, “provide the context (e.g., appearance, taste, texture/mouth-feel) for any of your ratings,” “provide context on satisfaction or boredom with individual foods, and variety of the food system overall,” and “provide any additional comments you’d like to share regarding this meal” to identify recurring patterns in data and group them into categories or themes. The first author, with consultation and code and theme review from the second author, coded the data. The reflexive thematic analysis was guided by the six steps outlined from Braun and Clarke (2006, 2025): (1) becoming familiar with dataset, (2) coding, (3) generating initial themes, (4) develop and reviewing themes, (5) refining, defining, and naming themes, and (6) summarizing results in writing. Given the novelty of a space exploration food system, we used an inductive method in that we specifically designed the survey questions to identify items related to variety, acceptability, and fatigue. We used a deductive and experiential approach to learn from the astronauts’ experience in the unique content of spaceflight, with both semantic and latent outcomes. We used these experiences to construct our ideas on strategies for exploration food systems.

## Results

**FIGURE 1:**
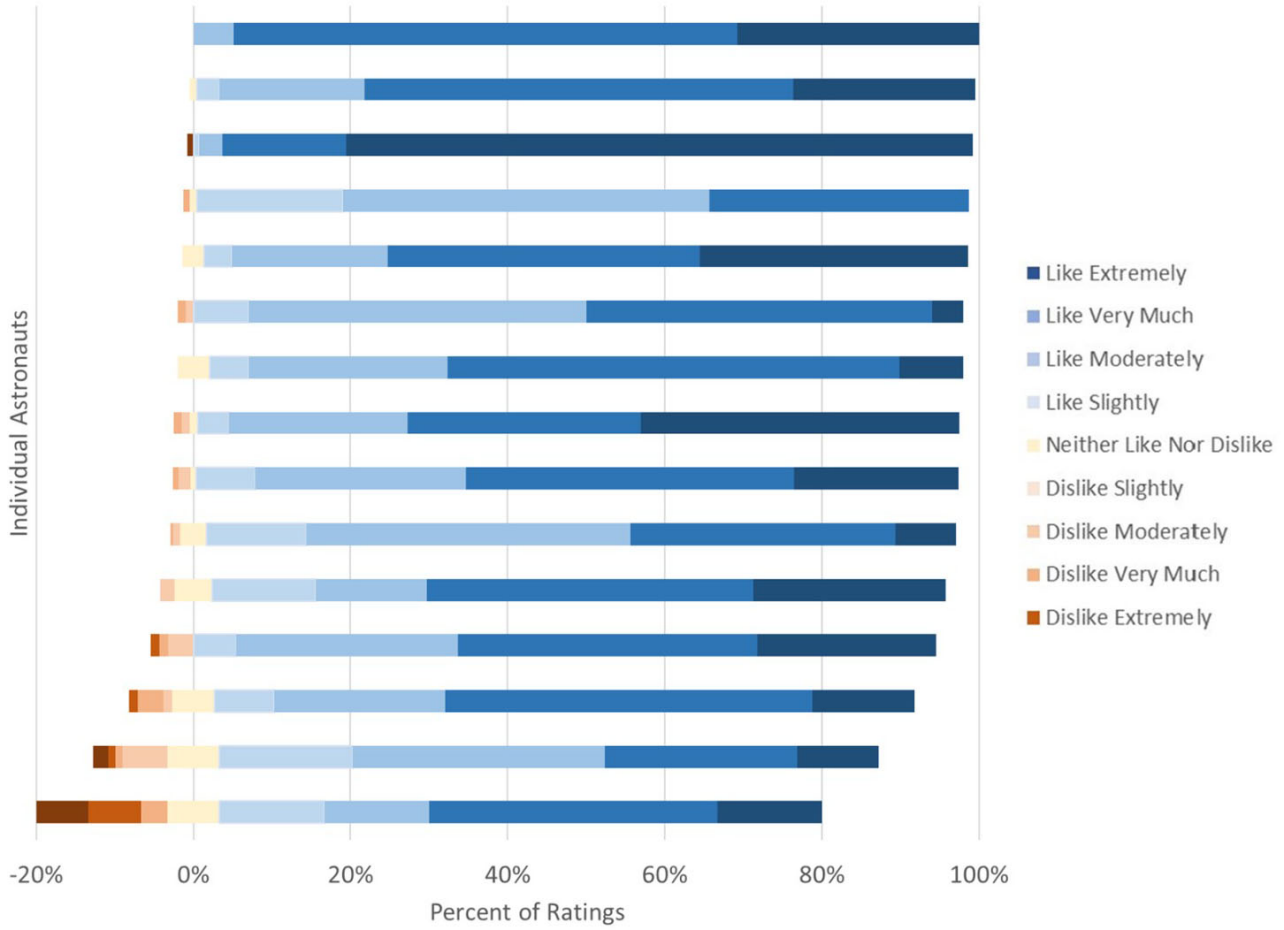
Food acceptability ratings by astronaut. Each bar represents one astronaut with colors representing the percentage of responses in each rating on the 9-pt hedonic scale (*n* = 15). Astronauts continued to score the foods they were choosing high throughout the mission, but comments indicated they were only selecting their favorites and not consuming other foods.

A total of 1,772 food scores for 434 different foods were received from all 15 astronauts. In contrast to the hypothesis, astronauts in this study did not show menu fatigue in relation to repeat consumption through decreasing food acceptability scores over time. Instead, there was no correlation between variety of foods an individual rated and how they rated foods overall (cor = 0.29, *p* = 0.2921). The food acceptability ratings by individual also were not related to mission length (cor = 0.26, *p* = 0.3528). Scores of liked foods did not decrease over time. Instead, higher scores were associated with a greater number of repeat scores (rmcorr = 0.1, *p* = 0.0018) although the effect was modest (0.07 ± 0.02, *p* = 0.0018 per additional one point rating from mixed modeling). This all supports a common debrief response, that astronauts limited selections to favorites early in the mission and continued to like those foods throughout the mission and score them acceptably (Figure 1).

**FIGURE 2:**
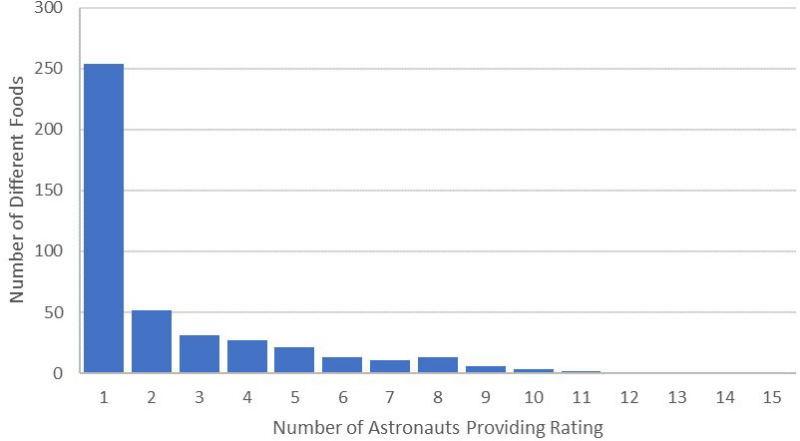
Number of different foods and number of astronauts who rated them. Each bar represents the number of foods scored by the corresponding number of astronauts. More than half of the foods were only scored by one astronaut, indicating the varying preferences across the astronauts and the importance of providing variety.

More than half of the foods rated (254 of 434) were only rated by one of the 15 astronauts (Figure 2). The number of different foods each astronaut rated was positively associated with mission length (cor = 0.87, *p* < 0.0001).

While many comment entries were left blank, some astronauts frequently provided comments. Comments to open-ended questions “provide context or boredom with individual foods, and variety of the food system overall” and “provide any additional comments you’d like to share regarding this meal” included both positive comments (e.g., aspects which helped to ward off menu fatigue or boredom) as well as negative comments (e.g., content of comments that indicated frustration, boredom, or onset of menu fatigue). Results of the thematic analysis suggested 7 themes: (1) importance of variety and when it was lacking, including indicators of onset of menu fatigue; (2) supplements to the menu that helped ward off menu fatigue; (3) components of food related to behavioral or physical health; (4) specific foods mentioned that were liked; (5) specific foods mentioned that were disliked, (6) ability to eat similarly to home, and (7) other factors related to food portioning and preparation impacting eating experience.

Comments indicating menu fatigue varied widely, with some astronauts indicating concerns about menu fatigue as early as 2 days into their missions and some astronauts never indicating they experienced fatigue (no fatigue comments). For the 9 astronauts with fatigue indicators in their comments, these first appeared between 1 and 4 months into their missions. During the debrief interviews astronauts indicated the fatigue did not impact the amount of food eaten over their mission length, however, several astronauts indicated that they thought it would on a longer mission or if no preference foods were available. In general, fatigue was due to individual expectations and tolerance for limited variety and the desire for preference foods. Comments further indicated that breakfast foods and vegetables were the most inadequate in variety and quantity.

We provide detail on each theme along with exemplar comments next.

**Theme 1: importance of variety and when it was lacking, including indicators of onset of menu fatigue**

Most, but not all, astronauts commented on the availability of different foods.

*We opened a few new BOBs* [food containers] *this weekend and were thinking it would be nice if there was some variety between BOBs. Even a few meals different would be great. It’s disheartening to open the same food week after week.* Astronaut A, 38% into mission.

Common subthemes were around the lack of variety in vegetables and a lack of protein in the breakfast foods. The acceptability in vegetables was also a recurring theme.

*There are not enough vegetables and fruit and these go fast. Too much nuts and too much soup, so we are always down to just soup and nuts (haha) because vegetables and fruit get eaten more quickly.* Astronaut D, 27% into mission.

Several times the need for more breakfast proteins was reported early on, even prior to menu fatigue.

*Overall, I am very happy with quantity and quality of the food system. I do wish there were more eggs in the standard breakfast menu.* … Astronaut E, 2% into mission.

Even though some liked the idea of cereal:

*Love having cereal for breakfast.* Astronaut F, 38% into mission.

**Theme 2: supplements to the menu that helped ward off menu fatigue**

While the lack of variety was often noted, crewmembers indicated that supplements to the menu helped keep a lack of variety from turning to boredom and menu fatigue. Supplemental food from the CSM, food augmentation for physiological studies, IP foods, condiments, fresh or specialty food deliveries with short shelf life that are only available with late load on resupply vehicles, and combining food in new ways were important to support consumption.

Many heavily relied on their preference foods from CSM for needed variety.

*Variety is good but supplement with CSM foods are needed to help with boredom.* Astronaut H, 13% into mission.

*The shrimp curry out of my CSM was a nice change from the standard menu. I have noticed that items are becoming a bit dull. Variety and new foods at this point in the mission is a most welcome addition.* Astronaut B, 45% into mission.

The majority of astronauts commented on the importance of IP food as a means of warding off menu fatigue.

*The variety of food from various space agencies is important. We should be able to select more food from ESA, JAXA, and CSA, even if there are not crewmembers flying with us.* Astronaut J, 25% into mission.

Astronauts also used strategies such as combining foods and suggested condiments as a means of warding off menu fatigue.

*Variety is okay, but at this point in the mission I find myself getting creative to change things up. Mixing together different foods, etc.* Astronaut F, 34% into mission.

*Almost everything could benefit from condiments. I wish there was greater variety in condiments, and more plentiful.* Astronaut J, 19% into mission.

**Theme 3: components of food related to behavioral or physical health**

Astronauts noted psychological or physical health aspects of food, or on one occasion how another astronaut’s diet may have had an impact to menu fatigue.

*These days I find myself full-ish, but not satisfied. I am definitely craving something, but not sure what. Variety? Salty crunchy food? More crunchy vegetables? A smoothie? Hard to say.* Astronaut L, 86% into mission.

*Boredom seems to be setting in*,… *Additionally, I have a crew member that is eating* [a specific diet]. *Because of* [astronaut’s] *food choices, we burn through many of the foods I like very quickly*…. *The perceived variety is reduced, and the repetition of food selections is increased. This specific diet by one crew member has an adverse affect on the rest of the crew.* Astronaut B, 92% into mission.

*Frustrating not enough healthy foods. Too much sugar. Not enough eggs.* Astronaut A, 56% into mission.

**Themes 4-5: Specific foods liked (Theme 4) or disliked (Theme 5)**

There were several comments that called out specific foods that were either liked or disliked, and sometimes a particular reason was given. The specific foods varied between astronauts and crews.

*Definitely need more variety. It is a gut punch to open a new food BOB and find it is the exact same as the last one. And, the foods that everyone dislikes we see … throw[n] away over and over* … Astronaut A, 49% into mission.

**Ability to eat similarly to home (Theme 6), and other factors related to food portioning and preparation impacting eating experience (Theme 7)**

Although commented on less frequently than other themes, several crewmembers commented about how the diet compared to what they would have at home, portion sizes being too large or too small, challenges with hydration of freeze-dried foods, and the importance of chilling some foods.

*The* [food] *is good, just super tiny. As with a lot of the portions, it is not nearly enough for a meal, more of a tease. If it was 3x the size, it would be just over [x] calories and constitute a meal.* Astronaut A, 63% into mission.

*Love using my crew preference spreads to make a toast type breakfast like at home.* Astronaut F, 61% into mission.

…*Orange juice is great, but it has to be cold. Very critical to have a Glacier to chill drinks. It makes a huge difference in acceptability*… Astronaut D, 16% into mission.

## Discussion

Data were collected from 15 astronauts on the ISS to determine the impact of repeat consumption on food acceptability within the current variety restrictions of the spaceflight food system and inform strategies to improve food system composition. The data did not support our hypothesis, that menu fatigue due to repeat consumption of foods within the variety restricted spaceflight food system led to decreases in acceptability of individual foods. Instead, the results indicated that astronauts limited selections to favorites early in the mission and continued to like those foods throughout the mission and score them acceptably.

Even with only one meal rated a week and approximately 200 items on the standard menu, more than 400 foods and beverages were rated. Although the personal preference provided to ISS astronauts is limited to only ∼20% of their options, it adds a significant variety for these crews. It is also important to note that we expect the variety consumed was much greater than what was indicated here, because the evaluation was limited to one meal per week. It is possible that 100 unique food items may be added by each individual’s CSM foods, adding a substantial variety to the ISS food system.

Despite the variety added by CSM foods in the current system, the majority of comments suggested the importance of variety and when it was lacking, including indicators of onset of menu fatigue (Theme 1). Comments were suggestive of the importance of variety and choice. Many crewmembers commented on the lack of variety in foods, particularly with vegetables and breakfast items. Comments indicated that some astronauts were craving foods that were not available and that although they rated what they consumed as acceptable, they could not find enough that they liked most to consume and stated that they were eating just to get nutrition. Over half of the astronauts provided comments indicating they were experiencing fatigue related to the menu as a whole. In a restricted system, limiting oneself to favorites could impact perception of the menu as a whole over time as well as total intake even if the subset of foods chosen for consumption is still considered acceptable.

Less than half of the foods and beverages were scored by multiple crewmembers (Figure 2), demonstrating varying preferences across astronauts and the importance of providing preference foods to support choice, and ultimately to support adequate intake, health, and performance. This was further reflected in the experiences of astronauts captured by Theme 2, of warding off menu fatigue with supplemental items from CSM or personal preference foods, international partner foods, condiments, and other ‘enhancements’. Importantly, many of these strategies may not be possible on exploration missions. First, food prepositioning timelines may occur well in advance of crew launch or any final crew changes, so use of preference foods may not align with those of the final crew. Second, although inclusion of IP foods increases variety, inclusion of these would need to be agreed upon between space agencies. Third, foods must fit within nutritional, safety, shelf life, and mass and volume requirements, and often supplemental foods are higher in salt and fat, have a shorter shelf life, or are packaged in heavier, bulkier packaging, such as cans, limiting their inclusion in the food system. Although some astronauts indicated a preference for the textures from canned foods over flexible food packages, mass and volume restrictions on future missions may not accommodate cans. Fortunately, many condiments do meet exploration requirements, and greater varieties of condiments have been included in the space food system. The variety increases if refrigeration is available for storage after the condiment is opened, but resource restrictions may also eliminate even a small chiller from many space missions.

Themes 4 and 5 focused on liked and disliked foods. Some crew comments indicated that foods that were disliked by an entire crew were thrown away. Excess food on spaceflight missions is extremely limited or not available, making it important to identify strategies to improve food system acceptability to ensure adequate intake and reduce waste. Because preferences varied widely between astronauts and between crews, removing and replacing foods to improve acceptability within a closed spaceflight system is not straightforward. Different astronauts may like and dislike the same foods. Food usage is currently tracked over time in spaceflight, and foods that are more commonly under-consumed or noted as disliked are replaced. Comments from Theme 1 indicate the most likely improvements would be to provide as much variety as possible, specifically increasing variety and quantity of fruits and vegetables, rotating foods, and modifying the breakfast foods to include more protein rich foods. Theme 3 comments, related to behavioral and physical health, such as evaluating sugar reduction, or identifying/developing crunchy foods that have a long shelf life and do not generate crumbs, may also be considered for potential improvements in the exploration food system strategy.

Theme 6 indicated that some astronauts were interested in finding foods that enabled them to eat like they do on Earth. This is a challenge in a restricted system given the great variety of options on Earth, and the varied preferences among crew. However, this idea supports the importance of provisioning whole, familiar foods. Although it is often assumed that as high-performing, highly-trained individuals, astronauts will eat anything required to complete a mission, food familiarity and quality assumes added psychological importance on extended-duration space missions, where it may mitigate stress associated with prolonged isolation and confinement (Stuster, 2010).

Theme 7 comments, on other factors impacting eating experience, varied among astronauts for different foods. Comments included where they would prefer a different portion size, the ability to easily hydrate foods to preference, or foods they would like to be able to hydrate longer or consume cold, both requiring refrigeration. Although all crew may not want to see a change in portion size, this is an area under consideration for a subset of exploration foods. Additionally, these comments highlight the importance of ensuring the exploration food preparation infrastructure is adequate as well as the foods. Although equipment for heating and hydration of foods, or chilling even a small number prior to consumption, can require significant mass, power, and volume, the comments here indicate that adequate preparation equipment is central to acceptability and consumption.

Understanding the relationship between food acceptability and mission duration may be even more important for exploration missions. Some future menu plans for highly resource-constrained missions limit astronauts to only the calories that they need, despite data that indicates that individuals who do not want to consume an item will lose weight rather than consume it (Sirmons et al., 2020). In addition, food may be prepositioned, limiting or eliminating the potential to provide preference foods that would not support late crew assignment changes. Since dietary intake is linked to health and performance (Douglas et al., 2022), inadequate food system design may have more severe health and performance consequences as mission duration and distance from Earth increase. These findings suggest that adequate acceptable variety may be critical to sustain intake on longer missions. Further data are needed in simulated or real long-duration contexts to define adequate variety and food system design requirements within the resource restrictions of longer missions. Another challenge to defining adequate variety and food system design was indicated in crew comments in this study (Theme 3). If a crewmember self-selects from only a few of the foods available, it may limit the availability of the foods they are consuming to other crewmembers due to the nature of a restricted, shared food system, which could have nutritional implications.

Data from this study, and future studies that include additional information on nutritional intake and body mass in relation to variety available and food acceptability over time, can be used to help design menus that are most acceptable for exploration crews. While many of the strategies used by astronauts on the ISS to increase variety and ward off menu fatigue, such as crew specific food items, may be limited or not available for exploration missions, some strategies noted here such as increasing variety and quantity in specific food groups, rotating foods, and ensuring adequate condiments may be possible. Future research can explore these and other approaches and inform the design of future exploration spaceflight food systems that allow us to sustain human health and performance as we push the boundaries of human space exploration.

## Data Availability

The datasets presented in this article are not readily available because data is available through NASA. Requests to access the datasets should be directed to https://nlsp.nasa.gov/explore/ page/home.
